# Relationship between energy balance and reward system gene polymorphisms and appetitive traits in young Mexican subjects

**DOI:** 10.3389/fnut.2024.1373578

**Published:** 2024-05-28

**Authors:** Ingrid Rivera-Iñiguez, Claudia Hunot-Alexander, Maricruz Sepúlveda-Villegas, Liliana Campos-Medina, Sonia Roman

**Affiliations:** ^1^Department of Genomic Medicine in Hepatology, Civil Hospital of Guadalajara, “Fray Antonio Alcalde”, Health Sciences Center, University of Guadalajara, Guadalajara, Jalisco, Mexico; ^2^Department of Human Reproduction Clinics, Infant Growth and Development, Institute of Human Nutrition, Health Sciences Center, Guadalajara, Jalisco, Mexico; ^3^Doctoral Program in Molecular Biology in Medicine, Health Sciences Center, University of Guadalajara, Guadalajara, Jalisco, Mexico

**Keywords:** polymorphisms, AEBQ, appetitive traits, young adults, personalized nutrition, tailoring, eating patterns

## Abstract

**Introduction:**

Appetitive traits are influenced by the interplay between genetic and environmental factors. This study aimed to explore the relationship between gene polymorphisms involved in the regulation of energy balance and food reward and appetitive traits in young Mexican subjects.

**Methods:**

This cross-sectional study involved 118 university freshman undergraduates who completed the Adult Eating Behaviour Questionnaire for Spanish speakers (AEBQ-Esp) to assess their appetitive traits. A real-time PCR system was employed to determine gene polymorphisms involved in energy balance (*LEP* rs7799039, *MC4R* rs17782313, *FTO* rs9939609, *GHRL* rs696217), and reward system (*DRD2/ANKK1* Taq1A rs1800497 and *COMT* rs4680).

**Results:**

The mean age of participants was 20.14 ± 3.95 years, 71.2% were women and their mean BMI was 23.52 ± 4.05 kg/m^2^. *COMT* Met allele carriers presented a significantly higher “Emotional overeating” mean score than Val allele carriers (2.63 ± 0.70 vs. 2.23 ± 0.70, *p* = 0.028). The *MC4R* CC + CT genotype correlated positively with “Emotional overeating” (Phi = 0.308, *p* = 0.01). The *COMT* MetMet+MetVal genotype correlated with higher “Emotional overeating” (*r* = 0.257, *p* = 0.028; Phi = 0.249, *p* = 0.033). The protective genotype *FTO* TT correlated positively with “Emotional undereating” (Phi = 0.298, *p* = 0.012). Carriers of the risk genotype *MC4R* CC + CT presented a higher risk of “Emotional overeating” than TT carriers (OR = 2.4, 95% CI 1.3–4.8, *p* = 0.034). Carriers of the risk genotype *COMT* MetMet+MetVal (OR = 3.4, 95% CI 1.1–10.3, *p* = 0.033), were associated with a higher risk of “Emotional overeating” than ValVal carriers. The protective *FTO* genotype TT was associated with “Emotional undereating” (OR = 1.8, 95% CI 1.1–9.1, *p* = 0.014).

**Discussion:**

The study found a relationship between the protective genotypes of *FTO* TT and “Emotional undereating” and risk genotypes of *COMT* Met/Met+Met/Val and *MC4R* CC + CT with “Emotional overeating.” These genetic factors may increase weight gain by enhancing hedonic food consumption and reducing satiety control. Future studies should focus on replication studies in ethnically diverse young adults and life stages to explore the relationship between polymorphisms and appetitive traits and weight. This will help tailor personalized nutrigenetic strategies to counteract disordered eating patterns leading to obesity and associated co-morbidities.

## 1 Introduction

Western societies and countries with emerging market economies such as Mexico are immersed in an environment conducive to the development of obesity (1). Given this rise, the “Behavioral Susceptibility Theory” (BST) proposes that some individuals are more sensitive to external food cues or “Food Responsiveness” and less responsive to internal cues for satiety or “Satiety Responsiveness,” therefore, increasing the risk of obesity (2, 3). The BST suggests that human body weight and appetitive traits are influenced by the interaction between genetic factors with the surrounding environment (4–6). “Food approach” and/or “food avoidance” appetitive traits, can be measured using psychometric questionnaires such as the Adult Eating Behavior Questionnaire (AEBQ) and its validated Spanish version (AEBQ-Esp) (7, 8). These traits are linked to higher body weight changes spanning from infancy to adulthood (5, 7).

Twin studies and Genome-Wide Association Studies (GWAS) have explained how genetic susceptibility to obesity is influenced by appetite; having a heightened sensitivity to internal hunger and fullness signals (“Satiety responsiveness”) and enjoying food (“Enjoyment of food”), as well as wanting to eat in response to the sight, smell, or taste of food (“Food responsiveness”), with heritability estimates of 63 and 75%, respectively (9). In the Gemini cohort, appetitive traits such as “Slowness in eating” (84%) and “Satiety responsiveness” (72%) were found to be highly genetically determined, while a moderate effect was associated with “Food responsiveness” (59%) and “Enjoyment of food” (53%) (10). Several extensive adult cohorts spanning the UK, the US, Canada, France, and Finland consistently reveal associations between genetic susceptibility and appetitive traits related to “Food responsiveness” such as “External eating,” “Uncontrolled eating” and “Disinhibition” (11–14). Notably, a longitudinal study of 2,464 British adults from the Whitehall II cohort demonstrated that “Disinhibition” mediated 34% of the association between a polygenic risk score (PRS) for obesity (comprising 97 genetic variants) in 20-year BMI trajectories during midlife (15).

Responsiveness to food cues, refers to the desire to eat when encountering food-related stimuli like the appearance, aroma, or taste of food (3). This aspect is linked to hedonic processes associated with pleasure and reward (16) regulated in the brain reward system. Gene polymorphisms related to the reward system such as the Dopamine Receptor 2 (*DRD2/ANKK1*) gene and Catechol-O-methyltransferase (*COMT*) seem to influence appetitive traits, by promoting hedonic food consumption and reducing satiety control (17). The A1 allele from the *DRD2/ANKK1 TaqIA1* (rs1800497) polymorphism contributes to decreased DRD2 receptor density and increases L-DOPA activity, impairing the reward system. The A1 allele is highly prevalent in Mexicans and contributes to detrimental dietary quality and metabolic disturbances (18). The *COMT* gene regulates emotional, cognitive, and appetitive processes (19, 20). The rs4680 (Val158Met) polymorphism in the *COMT* gene has been related to appetite conditioning (19).

Internal satiety signals such as hunger and fullness are regulated by hormones in the energy balance system (21). Three genes encode important components related to energy intake and energy expenditure balance such as Leptin (*LEP*), ghrelin (*GHRL*), melanocortin-4-receptor (*MC4R*), and the fat mass and obesity-associated gene (*FTO*) (22, 23). Leptin, the hormone that regulates fullness is codified by the Leptin (*LEP*) gene and the G allele from the −2548G > A, rs7799039 polymorphism of this gene has been linked with lower levels of postprandial fullness (24). The rs696217 polymorphism (Leu72Met) of the *GHRL* gene affects hunger and satiety. Individuals with the Met72Met genotype exhibit greater consumption of sugars and bread compared to carriers of the Leu72Leu genotype (25). There is evidence that individuals showing genetic risk factors exhibit varying scores related to “food approach” and “food avoidant” appetitive traits, as observed among 180 young Mexican subjects, where circulating IgG autoAbs reacting to ghrelin and leptin were evaluated (26). Additionally, *MC4R* regulates energy homeostasis and appetite signals (27, 28). The C allele from the −188 kb T > C, rs17782313 *MC4R* polymorphism is associated with higher BMI (29) and higher appetite (30). In a study with 221 obese Chilean children, the rs17782313 variant was associated with “Satiety responsiveness” and “Enjoyment of food” scores (31). This variant may be linked to childhood obesity, impacting factors such as “Enjoyment of food” and “Satiety responsiveness” and possibly “Eating in the absence of hunger” according to a case–control study involving 139 normal-weight and 238 obese children aged 6–12 years (32). The T > A, rs9939609 *FTO* gene polymorphism expressed in the hypothalamus, regulating food intake and energy expenditure is known to be related to higher BMI. Studies in children examining the relationship between appetitive traits and *FTO* reveal that those with the AA genotype are more prone to overweight or obesity. This inclination is attributed to lower “Satiety responsiveness,” heightened “Food responsiveness,” and increased “Enjoyment of food” in children and adolescents (33, 34).

Studies indicate that young adults, especially university students, undergoing a transitional life stage face challenges such as balancing work and study responsibilities, as well as managing stress (35). These stressors can adversely affect their eating behaviors, putting them at a heightened risk of developing disordered eating patterns (36, 37) and the development of obesity (38). However, little is known of the relationship that exists between different polymorphisms that participate in the regulation of food consumption and appetitive traits in young people, which could serve to tailor intervention in this age group and move toward a more personalized medicine and nutrition approach. Therefore, this study aimed to explore the relationship between polymorphisms of genes involved in energy balance (*LEP* rs7799039, *MC4R* rs17782313, *FTO* rs9939609, *GHRL* rs696217), and reward system (*DRD2/ANKK1* rs1800497 and *COMT* rs4680) and appetitive traits subscales of the AEBQ-Esp in young Mexican subjects.

## 2 Materials and methods

### 2.1 Participants and procedure

This study was carried out at the Department of Genomic Medicine in Hepatology from January 2018–2019. Due to the 2019 COVID pandemic, research activities were postponed and resumed from July to December 2022. The study involved primarily freshman undergraduates selected from the Health Sciences Center (*Centro Universitario de Ciencias de la Salud, CUCS*) at the Universidad de Guadalajara, a public non-fee-paying university. Students who did not have Spanish as their first language were not included in the research. Researchers initiated contact with university administrators, who, in turn, approached lecturers responsible for undergraduate courses, seeking their willingness to involve their classes in the study. The researchers secured informed consent from the students before commencing data collection. Ethical approval (number CI-08218) was obtained by the Institutional Review Board.

### 2.2 Measurement of appetitive traits

Appetitive traits were assessed using the AEBQ-Esp, a 30-item self-report questionnaire known for its validity and reliability in measuring seven distinct appetitive traits (8). The three “food approach” traits consisted of “Food responsiveness” (four items, e.g., I am always thinking about food), “Emotional overeating” (five items, e.g., I eat more when I’m angry), and “Enjoyment of food” (three items, e.g., I love food). Four “Food avoidance” traits included: “Satiety responsiveness” (four items, e.g., I get full up easily), “Emotional undereating” (five items, e.g., I eat less when I’m upset), “Food fussiness” (five items, e.g., I enjoy tasting new foods) and “Slowness in eating” (four items, e.g., I eat slowly). All participants responded to a 5-point Likert scale, ranging from 1 (“strongly disagree”) to 5 (“strongly agree”). Mean scores were calculated for each subscale and classified as dichotomous variables, as “high-score” for “food approach” traits if their score on each subscale was greater than three, likewise, they were classed as “low-score” for “food avoidance” traits if their score for each subscale was less than three.

### 2.3 DNA extraction and genotyping

A peripheral blood sample was obtained to perform the genomic DNA extraction from leukocytes with a modified Salting Out technique (39); a quantified concentration of 20 ng/μl with Nanodrop 2000c was stored at −70°C. A real-time PCR system was performed with predesigned TaqMan Single Nucleotide Polymorphism (SNP) Genotyping Assays (Applied Biosystems, Life Technologies, Camarillo, CA, USA) for the determination of *LEP* rs7799039 (C___1328079_10), *MC4R* rs17782313 (C__32667060_10), *FTO* rs9939609 (C__30090620_10), *GHRL* rs696217 (C___3151003_20), *DRD2/ANKK1* Taq1A rs1800497 (C___7486676_10) and *COMT* rs4680 polymorphisms (C__25746809_50) using a 96-well plate and then read on a Step One Plus thermocycler (Applied Biosystems). Thermal cycling conditions were the following: activation stage at 95°C for 10 min, denaturation stage of 40 cycles at 95°C for 15 s. And the annealing/extension stages at 60°C for 1 min.

### 2.4 Sociodemographic data

Participants reported their age, university, work status (full-time job; part-time job; self-employed; not working), marital status (not married; married; lives with partner), living status (at home; away from home), both parents’ work (full-time job; part-time job; self-employed; has different jobs; unemployed; does not have a father or mother) and both parents’ education (primary school; GCSE levels; A levels; bachelors; postgraduate). The latter information was used as a proxy for socioeconomic status (data not shown) (40).

### 2.5 Anthropometry

Height was measured without shoes to the nearest 0.1 cm (Seca stadiometer); weight was measured in kilograms to the nearest 100 g (Tanita weighing scale); and body mass index (BMI) was the ratio of weight (kg), by height (m^2^). BMI cut-off points were as follows: healthy weight (18.5–24.9 kg/m^2^) overweight (25–29.9 kg/m^2^), and obesity (>30 kg/m^2^).

### 2.6 Statistical analyses

The Kolmogorov–Smirnov test was applied to know the distribution of the analyzed data. Continuous variables were presented as mean ± standard deviations (SD), and categorical variables as percentages where appropriate. Cronbach’s alpha was performed to test internal reliability among the AEBQ-Esp subscales, considering an acceptable value of greater than 0.7. Comparisons were conducted using the T-test for normally distributed data and the Mann–Whitney *U* test for non-normally distributed data. Correlation analyses were conducted using point biserial correlations for normally distributed data, Kendall’s correlation for non-normally distributed data, and the Phi-Coefficient was also calculated. The association of genotypes with the subscales was estimated with an odds ratio (OR) test and a 95% confidence interval (CI). A *p*-value of <0.05 was considered significant. Statistical analysis was conducted with SPSS software (version 25.0; SPSS Inc., Chicago, IL, USA), significance level of *p* < 0.05. The Hardy–Weinberg equilibrium (HWE) was obtained with the Arlequin software for Windows (version 3.1; Berne, Switzerland).

## 3 Results

### 3.1 Population’s descriptive characteristics

Demographic, clinical, and genetic characteristics are presented in Table 1. In this study, 118 young adults (20.14 ± 3.95 years) were evaluated (71.2% women). The mean BMI was 23.52 ± 4.05 kg/m^2^.

**Table 1:** Population demographics and clinical characteristics (*n* = 118).

Characteristic	Mean ± SD
Age (years)	20.14 ± 3.95
Gender	
Female (n, %)	(84, 71.2)
Male (n, %)	(34, 28.8)
Weight (kg)	63.68 ± 14.14
BMI (Kg/m^2^)	23.52 ± 4.05
Underweight (n/%)	7 (5.9)
Healthy weight (n/%)	72 (61)
Overweight (n/%)	31 (26.3)
Obesity (n/%)	8 (6.8)

BMI, Body Mass Index.

Most subjects were healthy weight (61%), followed by 33.1% of excess weight, and 5.9% were underweight.

### 3.2 Descriptive data for appetitive traits and AEBQ-Esp internal validation

The means and standard deviations of the seven appetitive traits of the AEBQ-Esp subscales and their internal reliability are shown in Table 2. Cronbach’s alpha value >0.7 demonstrated good internal reliability in five of the seven traits, only the “Food fussiness” and “Slowness in eating” subscales were < 0.5.

**Table 2:** AEBQ-Esp subscales and internal reliability.

AEBQ-Esp subscales	Mean ± SD	Internal reliability (Cronbach’s alpha)
Food approach subscales		
Food responsiveness (FR)	2.99 ± 0.70	0.744
High (%)	55	
Low (%)	45	
Emotional overeating (EOE)	2.63 ± 0.78	0.779
High (%)	36	
Low (%)	64	
Enjoyment of food (EF)	4.21 ± 0.70	0.734
High (%)	96	
Low (%)	4	
Food approach subscales		
Satiety responsiveness (SR)	2.55 ± 0.77	0.748
High (%)	81	
Low (%)	19	
Emotional undereating (EUE)	3.07 ± 0.81	0.806
High (%)	53	
Low (%)	47	
Food fussiness (FF)	3.17 ± 0.60	0.423
High (%)	33	
Low (%)	67	
Slowness in eating (SE)	2.86 ± 0.70	0.518
High (%)	67	
Low (%)	33	

### 3.3 Allele and genotype frequencies of the analyzed gene polymorphism

The allele and genotype distribution of the analyzed gene polymorphisms are shown in Table 3. All analyzed polymorphisms were in Hardy–Weinberg equilibrium (HWE) (*p* > 0.05). A different number of samples were analyzed for each polymorphism. For the *MC4R,* the T allele was more prevalent in the population (88%), and the absence of the genotype CC was observed. The frequency of the G allele was higher in the genes *LEP*, *COMT*, and *DRD2*; also in these groups, the heterozygote (AG/GA) was the most frequent genotype. In the *FTO* gene, the frequency of the T allele was 70%, and 48% of homozygotes. In the case of the *GHRL* gene, the G allele had a frequency of 85%, while the TT genotype was present in only one subject. All analyzed polymorphisms were in Hardy–Weinberg equilibrium (HWE) (*p* > 0.05).

**Table 3:** Alleles and genotypes frequency of the gene polymorphisms.

Genes	n	HWE	Alleles n (%)		Genotypes n (%)		
*MC4R* T > C, (rs17782313)	69	0.276	T	C	TT	CT	CC
			122 (88)	16 (12)	53 (77)	16 (23)	0
*LEP* G > A, (rs7799039)	69	0.297	A	G	AA	AG	GG
			51 (39)	79 (61)	8 (12)	35 (54)	22 (34)
*FTO* T > A, (rs9939609)	71	0.653	A	T	AA	AT	TT
			39 (30)	93 (70)	5 (8)	29 (44)	32 (48)
*COMT* Val158Met, (rs4680)	93	0.419	Val	Met	ValVal	ValMet	MetMet
			116 (62)	70 (38)	38 (41)	40 (43)	15 (16)
*DRD2/ANKK1 Taq1A* (rs1800497)	61	0.868	A2	A1	A2A2	A2A1	A1A1
			76 (62)	46 (38)	24 (39)	28 (46)	9 (15)
*GHRL L*eu72Met, (rs696217)	109	0.302	G	T	GG	GT	TT
			186 (85)	32 (15)	78 (72)	30 (27)	1 (1)

HWE, Hardy Weinberg Equilibrium. Allele and genotype frequencies are expressed as percentage.

### 3.4 Appetitive traits according to gene polymorphisms

The means and SD of the AEBQ-Esp subscales according to the dominant model of the studied gene polymorphisms are presented in Table 4. There was a tendency toward carriers of the *MC4R* C allele to have lower “Emotional overeating” (2.42 ± 0.65, *p* = 0.08). Similarly, *LEP* G allele carriers (2.87 ± 0.65, *p* = 0.06) tended to present lower “Slowness in eating.” *FTO* carriers tended to have higher “Emotional undereating” (3.11 ± 0.79, *p* = 0.061). Met allele carriers from the *COMT* gene tended to present higher “Food fussiness” (3.40 ± 0.37, *p* = 0.06) and presented significantly higher “Emotional overeating” (2.63 ± 0.70, *p* = 0.028) than Val allele carriers. No other significant differences were found.

**Table 4:** Appetitive traits according to gene polymorphisms.

AEBQ	*MC4R*		*LEP*		*FTO*		*COMT*		*DRD2/ANKK1*		*GHRL*	
	TT	CC + CT	AA	GG + AG	TT	AA + AT	ValVal	MetMet + MetVal	A2A2	A1A1 + A1A2	GG	TT + GT
FR	2.98 ± 1.03	2.95 ± 0.68	2.96 ± 0.62	2.95 ± 0.79	2.94 ± 0.69	2.99 ± 0.78	2.96 ± 0.69	2.93 ± 0.79	2.89 ± 0.74	2.88 ± 0.75	3.00 ± 0.71	2.98 ± 0.71
EOE	2.78 ± 0.91	2.42 ± 0.65*	2.46 ± 0.67	2.52 ± 0.74	2.42 ± 0.73	2.55 ± 0.73	2.26 ± 0.70	2.63 ± 0.70**	2.34 ± 0.67	2.48 ± 0.73	2.65 ± 0.81	2.60 ± 0.73
EF	4.02 ± 0.82	4.19 ± 0.73	4.13 ± 0.77	4.12 ± 0.77	4.04 ± 0.77	4.24 ± 0.72	4.16 ± 0.69	4.11 ± 0.79	4.01 ± 0.66	4.11 ± 0.81	4.1 ± 0.67	4.19 ± 0.77
SR	2.78 ± 0.80	2.71 ± 0.75	2.91 ± 0.92	2.63 ± 0.65	2.82 ± 0.78	2.63 ± 0.72	2.84 ± 0.87	2.69 ± 0.69	2.96 ± 0.67	2.63 ± 0.79	2.54 ± 0.78	2.60 ± 0.81
EUE	3.26 ± 0.88	3.05 ± 0.80	3.26 ± 0.86	3.06 ± 0.77	3.15 ± 0.62	3.11 ± 0.79*	3.24 ± 0.98	3.10 ± 0.66	3.23 ± 0.82	3.16 ± 0.82	3.06 ± 0.83	3.22 ± 0.77
FF	3.38 ± 0.34	3.30 ± 0.36	3.36 ± 0.29	3.30 ± 0.39	3.31 ± 0.32	3.34 ± 0.38	3.24 ± 0.29	3.40 ± 0.37 *	3.43 ± 0.34	3.26 ± 0.36	3.16 ± 0.59	3.12 ± 0.66
SE	3.04 ± 0.74	2.99 ± 0.61	3.16 ± 0.56	2.87 ± 0.65*	3.04 ± 0.56	2.93 ± 0.66	2.93 ± 0.62	3.03 ± 0.63	2.99 ± 0.55	2.94 ± 0.68	2.86 ± 0.74	2.82 ± 0.58

FR, Food responsiveness; EOE, Emotional overeating; EF, Enjoyment of food; SR, Satiety responsiveness; EUE, Emotional undereating; FF, Food fussiness; SE, Slowness in eating. Results are presented as means and standard deviations. Comparisons were conducted using the T-test for normally distributed data and the Mann–Whitney U test for non-normally distributed data (EF, EUE and GHRL). ***p < 0.01, **p < 0.05, *p < 0.1.

### 3.5 Correlations between gene polymorphisms and appetitive traits

The correlation analysis between genetic models and mean appetitive trait values is listed in Table 5, and correlations between gene polymorphisms and appetitive traits as dichotomous variables can be found in Table 6. In terms of “food approach” subscales, there was a tendency toward a positive correlation between the *MC4R* CC + CT genotype and “Emotional overeating” mean scores (*r* = 0.211, *p* = 0.082, Table 5), as well as with the categorical variable (Phi = 0.308, *p* = 0.010, Table 6). Similarly, the *COMT* MetMet+MetVal genotype correlated with higher “Emotional overeating” mean scores (*r* = 0.257, *p* = 0.028; Phi = 0.249, *p* = 0.033). Carriers of the higher risk genotype MetMet+MetVal were positively correlated with all “food approach” traits (Phi = 0.378, *p* = 0.002, Table 6). There was a tendency toward a correlation of the high-risk genotype *LEP* GG + AG with higher “Enjoyment of food” mean scores (*r* = 0.209, *p* = 0.082, Table 6).

**Table 5:** Correlations between gene polymorphisms and appetitive traits mean values.

Genetic dominant models	FR mean	EOE mean	EF mean	SR mean	EUE mean	FF mean	SE mean
*MC4R* TT vs. CC + CT	0.011	0.211*	0.069	−0.038	−0.066	−0.087	−0.31
*LEP* AA vs. GG + AG	0.012	0.022	0.019	0.209*	0.160	0.166	0.225
*FTO* TT vs. AA + AT	0.036	0.089	0.129	−0.123	−0.190*	0.055	−0.088
*COMT* MetMet + MetVal vs. ValVal	0.015	0.257**	0.004	−0.096	−0.063	0.216*	0.076
*DRD2/ANKK1* A1A1 + A1A2 vs. A2A2	0.001	0.097	0.092	−0.213*	−0.047	−0.235*	−0.042
*GHRL* GG vs. TT + GT	0.068	0.002	0.026	−0.006	−0.070	−0.020	0.047

FR, Food responsiveness; EOE, Emotional overeating; EF, Enjoyment of food; SR, Satiety responsiveness; EUE, Emotional undereating; FF, Food fussiness; SE, Slowness in eating. Correlation analyses were conducted using point biserial correlations for normally distributed data and Kendall’s correlation for non-normally distributed data (EF, EUE and GHRL). ***p < 0.01, **p < 0.05, *p < 0.1.

**Table 6:** Correlations between gene polymorphisms and appetitive traits as dichotomous variable.

Genetic dominant models	Food approach (FA)				Food avoidance (FV)				
	FR cat	EOE cat	EF cat	FA cat	SR cat	EUE cat	FF cat	SE cat	FV cat
*MC4R* TT vs. CC + CT	0.077	0.308**	0.158	1.22	−0.014	0.093	0.014	−0.018	0.082
*LEP* AA vs. GG + AG	0.011	0.020	0.209*	0.378**	−0.204	−0.212	−0.157	−0.083	0.052
*FTO* TT vs. AA + AT	0.068	0.042	0.017	0.055	0.107	0.298**	0.089	0.054	−0.063
*COMT* MetMet + MetVal vs. ValVal	0.129	0.249**	0.072	0.087	0.164	0.085	−0.120	−0.100	0.089
*DRD2/ANKK1* A1A1 + A1A2 vs. A2A2	0.035	0.117	0.196	0.016	0.168	0.005	0.165	−0.008	0.042
*GHRL* GG vs. TT + GT	0.079	0.026	0.153	−0.027	−0.013	0.067	−0.046	−0.029	0.079

FR, Food responsiveness; EOE, Emotional overeating; EF, Enjoyment of food; SR, Satiety responsiveness; EUE, Emotional undereating; FF, Food fussiness; SE, Slowness in eating. Correlation analyses were conducted using Phi-Coefficient. ***p < 0.01, **p < 0.05, *p < 0.1.

As part of the relationships between gene polymorphism and “food avoidant” traits, we found that the protective genotype *LEP* AA was positively correlated with “Satiety responsiveness” (*r* = 0.209, *p* = 0.085). In contrast, there was a tendency toward a negative correlation between the risk genotype *DRD2* A1A1 + A1A2 with lower “Satiety responsiveness” (*r* = −0.213, *p* = 0.08). The high-risk genotype *FTO* AA+AT was negatively correlated to “Emotional undereating”; however, it was not significant (*r* = −0.190, *p* = 0.061). There was a significant positive correlation between the protective *FTO* genotype with “Emotional undereating” as a categorical variable (Phi = 0.298, *p* = 0.012). In this study there were only correlation tendencies with “Food fussiness” and gene polymorphisms; *COMT* high-risk genotype MetMet+MetVal and “Food fussiness” (*r* = 0.216, *p* = 0.066), and the protective genotype *DRD2* A2A2 (*r* = −0.235, *p* = 0.60).

### 3.6 Association between gene polymorphisms and appetitive traits

Carriers of the risk genotype *MC4R* CC + CT presented a higher risk of “Emotional overeating” than TT carriers (OR = 2.4, 95% CI 1.3–4.8, *p* = 0.034). Similarly, carriers of the risk genotype *COMT* MetMet+MetVal, were associated with a higher risk of “Emotional overeating” than Val carriers (OR = 3.4, 95% CI 1.1–10.3, *p* = 0.033). The protective genotype TT from *FTO* was associated with higher “Emotional undereating” (OR = 1.8, 95% CI 1.1–9.1, *p* = 0.014) (Data not shown).

## 4 Discussion

Mexico presents one of the highest rates of obesity worldwide, and there is limited data in the Mexican population related to appetitive traits and genetic risk factors that contribute to obesity. This appears to be the first study that explores the relationship between gene polymorphisms related to energy balance, hunger, satiety, and reward with scores of “food approach” and “food avoidant” appetitive trait subscales in young healthy weight (BMI: 23.52 ± 4.05) Mexican adults. To determine the appetitive traits in this population, the AEBQ-Esp was used, which has previously been validated in the Mexican population (8). In this study, the results reveal that the AEBQ-Esp with the exclusion of the “Food fussiness” and “Slowness in eating” subscales, were internally reliable (0.734–0.906); therefore, we recommend care be taken with regards to results interpreted using these two subscales. These results differ from the original validation of the AEBQ-Esp, where alpha Cronbach values were 0.70 and 0.91, respectively, for these subscales (8). Lower internal reliability values have also been found when mothers of children (41) and toddlers (42) complete the AEBQ-Esp questionnaire, in samples of Mexican subjects. This could be due to the size of the population obtained in this sample.

In the present study, we found important relationships between the analyzed gene polymorphisms with scores of “food approach” (“Emotional overeating” and “Enjoyment of Food”) and “food avoidant” (“Satiety responsiveness,” “Food fussiness” and “Emotional undereating”) appetitive traits. In terms of “Emotional overeating,” *MC4R* has been related to obesity by disrupting energy homeostasis and appetite signals (27, 28). Specifically, the rs17782313 polymorphism of *MC4R* has been previously associated with higher BMI and higher appetite (30). Moreover, when C carriers are exposed to unhealthy eating patterns, the risk of developing depressive feelings increases (43). Our analysis revealed a tendency toward a correlation between the *MC4R* (rs17782313) CC + CT genotype and “Emotional overeating” mean value (*r* = 0.211, *p* = 0.082) and as a categorical variable (Phi = 0.308, *p* = 0.010). Moreover, carriers of the risk genotype *MC4R* CC + CT presented a higher risk of “Emotional overeating” than TT carriers (OR = 2.4, 95% CI 1.3–4.8, *p* = 0.034). This is similar to what was found in an Iranian cross-sectional study in adults overweight and obese, where obese individuals carriers of the C risk allele of *MC4R* (rs17782313) exhibited a higher mean score of “Emotional eating” assessed with the self-report Emotional Eating Questionnaire (EEQ). In that study, *MC4R* rs17782313 was also associated with appetite, energy intake, nutrients, stress, ghrelin, and cortisol levels (44). In contrast, in a study with adults with obesity from Chile, “Uncontrolled eating” and “Emotional eating” measured using the Three-Factor Eating Questionnaire-R18, showed that carriers of the risk allele rs17782313 presented higher scores of “Emotional undereating” compared to non-carriers (45).

The *COMT* gene plays a key role in the reward system, where it regulates emotional, cognitive, and appetite processes (19, 20). Moreover, it is important to highlight that in the Mexican population, a higher prevalence of the Met allele (63%) has been reported (46), which contrasts with the prevalence in other populations. The Met allele has been reported to be associated with a personality trait such as “worrier” and with impulsivity and negative emotions (47, 48). In line with this, in the present study, we found that the high-risk genotype *COMT* (rs4680) MetMet+MetVal was positively correlated with the mean scores (*r* = 0.257, *p* = 0.028), and categorical variables of “Emotional overeating” (Phi = 0.249, *p* = 0.033). In addition, we only found that carriers of the higher risk genotype MetMet+MetVal were positively correlated with “food approach” traits (Phi = 0.378, *p* = 0.002). In contrast, in a cross-sectional study of 3-4-year-old children conducted in Brazil, results showed that the *COMT* Val158Met polymorphism contributed to a higher intake of palatable food. That study revealed that carriers of the Val allele presented a higher intake of fats when compared to MetMet homozygotes (49).

In terms of “food avoidant” traits, we found that the protective genotype *LEP* AA was positively correlated with “Satiety responsiveness” (*r* = 0.209, *p* = 0.085) and a tendency toward a negative correlation between the risk genotype *DRD2* A1A1 + A1A2 and “Satiety responsiveness” (*r* = −0.213, *p* = 0.08). Valladares found that carriers of the class II allele from the non-translatable hypervariable region at the 3′ end of the gene *LEP* (TTTC)n, had higher scores of “Slowness in eating” vs. non-carriers (31). It has been reported previously, that rs7799039 is associated with higher consumption of energy intake (50). These studies so far, highlight that there is a need for more studies to determine whether polymorphisms of *LEP* could be influencing emotional overeating.

Moreover, in this study, we found a tendency toward a negative correlation between the high-risk genotype *FTO* AA+AT and “Emotional undereating” (*r* = −0.190, *p* = 0.061). There was a significant positive correlation between the protective genotype from the *FTO* with “Emotional undereating” as a categorical variable (Phi = 0.298, *p* = 0.012). The protective *FTO* genotype TT was associated with higher “Emotional undereating” (OR = 1.8, 95% CI 1.1–9.1, *p* = 0.014). In contrast, Valladares and colleagues, in their study conducted among Chilean children with obesity, did not find any relationships between “Emotional undereating” and the genetic polymorphisms studied in that population (31).

On the other hand, “Food fussiness” is a “food avoidance” trait, that refers to the reluctance to eat both new and familiar foods, pickiness of the flavor and texture of foods, and it has been mostly explored among children (51, 52). Interestingly, although not significant, in this study, we found a tendency toward the presence of the *COMT* high-risk genotype MetMet+MetVal and higher “Food fussiness” (*r* = 0.216, *p* = 0.066), and the protective genotype *DRD2* A2A2 with lower “Food fussiness” (*r* = −0.235, *p* = 0.60). However, care should be taken not to put too much weight on these results, as the “Food fussiness” “subscale was not reliable.” The genes that contribute to “Food fussiness” have not been identified yet. However, it has been previously reported that “Food fussiness” is highly heritable (53). Potentially a larger sample size study that explores the role of the *COMT* and *DRD2* genes and “Food fussiness” would be necessary. According to data from the Gemini twin study, genetic correlations have been observed between “Food fussiness” and preferences for vegetables and fruits (54). It would be worth analyzing if the *COMT* and *DRD2* genes influence the intake of fruits and vegetables in a larger population.

Finally, in the context of the global obesity epidemic including in Mexico, these results show the importance of moving toward a more personalized medicine and nutrition approach, toward assessing risk, and finding novel ways to prevent and treat diseases (55). Furthermore, the presence of the respective risk alleles among the studied genes underscores the need for preventive strategies against further weight gain among the population (56). In the present study, 61% of participants presented healthy weight and the prevalence of overweight and obesity was 33.1%. Therefore, a future perspective could be including a bigger sample size to perform analysis according to participant’s BMI. Also, appetitive traits can be targeted to create behavioral strategies for weight reduction when personalized profiles are generated for individuals (57). Also, appetitive traits reflect the relationship that individuals have with food and response to food cues in the environment, which initiate in childhood and continue until adolescence as a risk factor for the development of eating disorders (58) is a further complication in this age group.

Our results need to be considered in view of several limitations. We recognize that in a cross-sectional study, it is complex to infer causal relationships because it only involves a one-time measurement, and we did not consider previous individual environmental factors that might influence appetitive traits including the 2019 COVID pandemic. Similarly, the pandemic affected our ability to conduct research activities, but we resumed activities and finalized the data analysis in 2022. Young adults may tend to under-report certain traits perceived as less desirable, such as higher “Food responsiveness,” while potentially over-reporting traits considered more favorable, like higher “Satiety responsiveness.” Conversely, it’s worth noting that social desirability tends to increase with age and be more prevalent in females (59). In addition, in this study, we had higher participation of female than male students. These young adults were recruited from the Health Sciences Center at the Universidad de Guadalajara, in which more than 60% of the students are females, specifically students enrolled in Nutrition are within a healthy BMI. We recognize that the characteristics and size of this sample may limit generalizing the results and detecting further associations. Including more male participants may allow us to identify associations related to gender. However, it has been suggested that certain appetitive traits influence young females to be more susceptible to develop overweight or obesity (60). We also did not evaluate physical activity levels which seems to have a modifying effect on certain SNPs such as the rs993609 *FTO* polymorphism (61, 62) and it is possible that individuals who engage in higher physical activity levels could have lesser levels of stress or negative emotions that influence emotional overeating. Despite the limitations of this study, it is important to recognize that young university adults experience a drastic transition in their lifestyles, which together with their genetic background may influence certain appetitive traits that could increase the risk of overweight and obesity. Multiple environmental factors and genes are involved in eating behavior, so there is a need to continue to expand the number of genes related to energy balance, appetite, satiety, and food reward and conduct longitudinal studies in different populations.

## 5 Conclusion

In this study the risk genotypes of *COMT* Met+Met and *MC4R* TT correlated with higher “Emotional overeating” and the protective *FTO* genotype TT was also associated with higher “Emotional undereating.” These results may facilitate understanding the genetic influence of weight gain by promoting hedonic food consumption and reductions in satiety regulation and could signal the need for more tailored strategies, including personalized medicine, personalized nutrition, or behavior-based treatment strategies that can help improve eating behaviors in young subjects. Similarly, identifying individuals at a higher risk of presenting appetite traits that facilitate cravings and food overconsumption could help prevent the development of obesity at earlier stages, even starting as early as childhood. These results may also help decision-makers to create public health recommendations and policy changes related to environmental factors that could be triggers of these high-risk appetitive traits. These findings require replication in different culturally diverse young adults, as well as in different stages of the life course. Future studies should also examine the prospective relationship between polymorphisms and appetitive traits and explore how these associations may relate to changes in weight and how engagement in healthy lifestyle behaviors could help modulate these associations.

## Data availability statement

The original contributions presented in the study are included in the article/supplementary material, further inquiries can be directed to the corresponding author.

## Ethics statement

The studies involving humans were approved by Comités de Investigación, Ética en Investigación y Bioseguridad del Centro Universitario de Ciencias de la Salud, Universidad de Guadalajara. The studies were conducted in accordance with the local legislation and institutional requirements. The participants provided their written informed consent to participate in this study.

## Author contributions

IR-I: Conceptualization, Project administration, Resources, Visualization, Writing – review & editing, Formal analysis, Funding acquisition, Investigation, Writing – original draft. CH-A: Conceptualization, Formal analysis, Funding acquisition, Investigation, Project administration, Visualization, Writing – original draft, Writing – review & editing, Resources. MS-V: Formal analysis, Investigation, Visualization, Writing – review & editing. LC-M: Formal analysis, Investigation, Visualization, Writing – review & editing. SR: Conceptualization, Formal analysis, Funding acquisition, Investigation, Project administration, Resources, Supervision, Visualization, Writing – review & editing.
